# Lack of an Association between Two BER Gene Polymorphisms and Breast Cancer Risk: A Meta-Analysis

**DOI:** 10.1371/journal.pone.0050857

**Published:** 2012-12-14

**Authors:** Bian Wu, Hong-Li Liu, Sheng Zhang, Xiao-Rong Dong, Gang Wu

**Affiliations:** 1 Cancer Center, Union Hospital, Tongji Medical College, Huazhong University of Science and Technology, Wuhan, China; University of Porto, Portugal

## Abstract

**Background:**

The base excision repair (BER) pathway removes DNA damage caused by ionizing radiation, reactive oxidative species and methylating agents. ADPRT and APE1 are two important genes in the BER pathway. Several studies have evaluated the association between polymorphisms in the two BER genes (ADPRT Val762Ala and APE1 Asp148Glu) and breast cancer risk. However, the results are inconsistent.

**Methodology/Principal Findings:**

In this study, we conducted a meta-analysis to derive a more precise estimation. A total of 8 studies were included in the meta-analysis (6 studies with 2,521 cases and 2,652 controls for ADPRT Val762Ala polymorphism and 5 studies with 2,539 cases and 2,572 controls for APE1 Asp148Glu polymorphism). For ADPRT Val762Ala polymorphism, no obvious associations were found for all genetic models (Val/Ala vs. Val/Val: OR = 0.960, 95% CI = 0.845–1.090; Ala/Ala vs. Val/Val: OR = 0.897, 95% CI = 0.683–1.178; dominant model: OR = 0.953, 95% CI = 0.843–1.077; and recessive model: OR = 1.084, 95% CI = 0.838–1.403). For APE1 Asp148Glu polymorphism, also no obvious associations were found for all genetic models (Asp/Glu vs. Asp/Asp: OR = 0.947, 95% CI = 0.829–1.082; Glu/Glu vs. Asp/Asp: OR = 0.958, 95% CI = 0.813–1.129; dominant model: OR = 0.946, 95% CI = 0.835–1.072; and recessive model: OR = 1.004, 95% CI = 0.873–1.155). In the subgroup analysis by ethnicity or study design, still no obvious associations were found.

**Conclusions/Significance:**

This meta-analysis indicates that ADPRT Val762Ala and APE1 Asp148Glu polymorphisms are not associated with increased breast cancer risk.

## Introduction

Breast cancer is currently the most common cancer and one of the main causes of cancer-related death in the world, which has become a major public health challenge [1]. It is a multifactorial disease caused by complex genetic and environmental factors [2]. Genetic variation in DNA repair genes can cause altered DNA repair function, resulting in accumulation of DNA damage, followed by cell apoptosis or unregulated cell growth and cancer. Individual variations in DNA damage and repair have been associated with breast cancer susceptibility and highlight the importance of DNA damage/repair in the development of the disease. Among DNA repair systems, the base excision repair (BER), which is an important pathway responsible for the repair of base damage and single strand breaks caused by X-rays, oxygen radicals, and alkylating agents, has been associated with risk of cancers [3]–[6].

The BER pathway consists of at least 11 DNA damage specific glycosylases and more than 20 further proteins [7]. Two of the most important proteins are adenosine diphosphate ribosyl transferase (ADPRT) and apurinic/apyrimidine endouclease 1 (APE1). ADPRT, also called poly (adenosine diphosphate-ribose) polymerase-1 (PARP-1), specifically binds to DNA strand breaks and recruits XRCC1-Lig3α complex, which is crucial to stimulating and executing the BER pathway [8], [9]. APE1 is the rate-limiting enzyme in the BER process and responsible for the repair of DNA and protecting cells against the effect of endogenous and exogenous agents [6], [10]. It cleaves 5 of DNA abasic sugar residues generated from exogenous factors, such as ionizing radiation and environmental carcinogens, as well as endogenous agents from normal cellular metabolism [11].

Several original studies have investigated the association between ADPRT Val762Ala and APE1 Asp148Glu polymorphisms and risk of breast cancer, but the results remain inconsistent, partially due to insufficient power in each of published studies which have been based on relatively small sample sizes. To explore a more precise estimation of the association between the two polymorphisms and risk of breast cancer, a meta-analysis was performed.

## Methods

### Search strategy

A literature search of Pubmed and Embase (updated to 2011/08/01) was conducted without a language limitation, using the following keywords and subject terms: “ADPRT or PARP1”, “APE1 or APEX1”, “polymorphism”, and “breast”. All searched studies were retrieved, and their bibliographies were checked for other relevant publications. Review articles and bibliographies of other relevant studies identified were hand-searched to find additional eligible studies. Only published studies with full text articles were included. When more than one of the same patient population was included in several publications, only the most recent or complete study was used in this meta-analysis. If necessary, we attempted to contact the corresponding authors of retrieved articles to acquire additional information.

### Inclusion criteria

The following criteria were used for the study selection: (1) evaluation of the polymorphism and breast cancer risk; (2) study designed as case-control; and (3) sufficient published data for calculating odds ratios (OR) with their 95% confidence interval (95% CI).

### Date extraction

Information was carefully extracted from all eligible publications independently by two investigators according to the inclusion criteria listed above. For conflicting evaluation, an agreement was reached following discussion. For each study, the following characteristics were collected: first author's name, year of publication, ethnicity, study design (control source and matching), genotyping results of cases and controls. We did not define any minimum number of patients to include in our meta-analysis.

### Statistical analysis

A statistical test for heterogeneity was performed based on the Q test and I^2^ test. If the P value is greater than 0.10 for the Q test which indicates a lack of heterogeneity among studies, the pooled OR estimate of the each study was calculated by the fixed-effects model (the Mantel–Haenszel method) [12]. Otherwise, the random-effects model (the DerSimonian and Laird method) was used [13]. The value of the I index is used to assess the degree of heterogeneity (I^2^<25%: no heterogeneity; 25%<I^2^<50%: moderate heterogeneity; 50%<I^2^<75%: high heterogeneity; I^2^>75%: extreme high heterogeneity). Subgroup analyses were performed by ethnicity and study design. Sensitivity analyses were also performed to identify the influence of the individual studies on the combined OR. Hardy-Weinberg equilibrium (HWE) was tested by the χ^2^ (P<0.05 was considered representative of statistical significance). The minor allele frequency (MAF) was also calculated for the controls. Publication bias was assessed by performing funnel plots qualitatively, and estimated by Egger's test (P<0.1 was considered representative of statistical significance) [14]. All the statistical analyses were done using STATA version 11 (StataCorp LP, College Station, Texas, USA).

## Results

### Study characteristics

**Figure 1:**
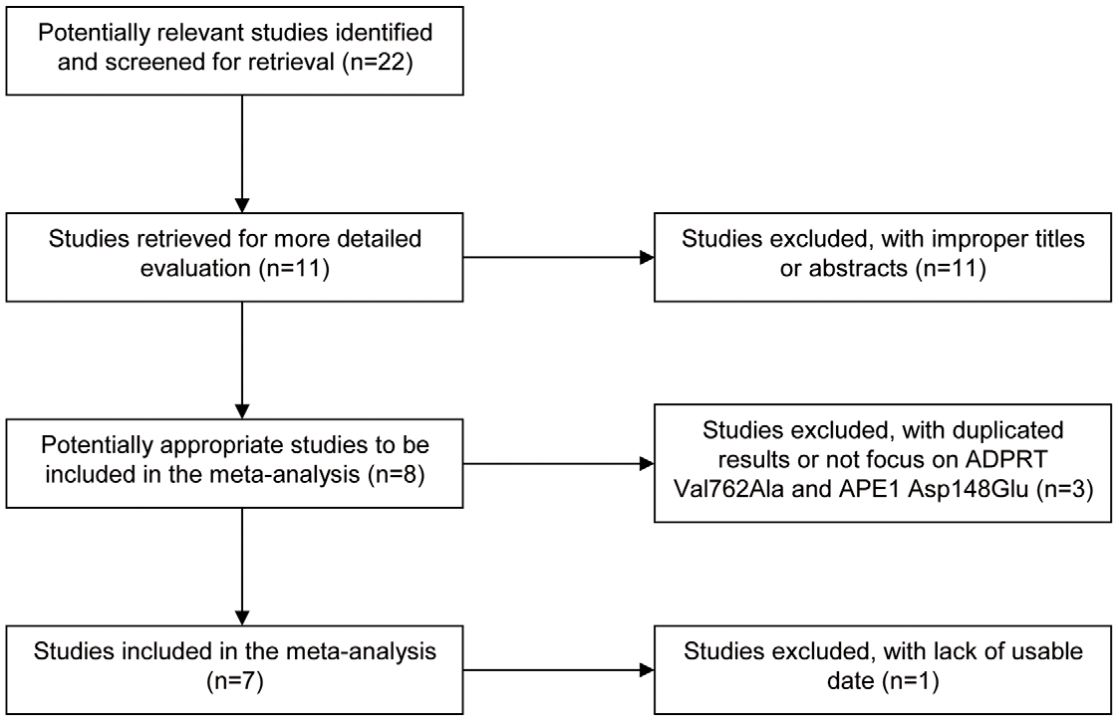
Flow diagram of study identification.

A total of 22 articles were achieved by literature search from PubMed and EMBASE. As shown in Figure 1, 11 eligible studies were retrieved for detailed evaluation. We excluded four studies (two with duplicated results, one not focus on ADPRT Val762Ala and APE1 Asp148Glu, and one with lack of usable data). Finally, a total of 7 studies fulfilling the inclusion criteria were identified [15]–[21]. In one of these studies, the genotype frequencies were presented separately according to Caucasian study and African-American study, and thus each study in the literature was considered separately for meta-analysis. Therefore, a total of 8 studies were included in the meta-analysis with 2521 cases and 2652 controls for ADPRT Val762Ala polymorphism and with 2539 cases and 2572 controls for APE1 Asp148Glu polymorphism. The studies identified and their main characteristics are summarized in Table 1 and Table 2. All studies indicated that the distribution of genotypes in controls was in agreement with HWE and the minor allele frequencies (MAFs) were also calculated for the controls (all were greater than 0.05 except one group) (Tables 2).

**Table 1:** Characteristics of studies included in the meta-analysis for ADPRT and APE1.

First author	Year	Country	Ethnicity	Control source	Matching	Cases	Controls
ADPRT							
Yawei Zhang	2006	USA	Caucasian	healthy	age	1716	1371
Xiangjun Zhai	2006	China	Asian	healthy	age	302	639
Wen-hui Cao	2007	France	Caucasian	healthy	—	83	100
Francine Durocher	2007	Canada	Caucasian	healthy	—	54	73
Tasha R.Smith	2008	USA	Caucasian	healthy	age and ethnicity	314	397
Tasha R.Smith	2008	USA	African-American	healthy	age and ethnicity	52	72
APE1							
Yawei Zhang	2006	USA	Caucasian	healthy	age	1529	1207
Suleeporn Sangrajrang	2008	Thailand	Asian	healthy	—	507	425
Tasha R.Smith	2008	USA	Caucasian	healthy	age and ethnicity	319	405
Tasha R.Smith	2008	USA	African-American	healthy	age and ethnicity	53	75
K.Jelonek	2010	Poland	Caucasian	healthy	age	91	412

**Table 2:** Distribution of ADPRT and APE1 genotype among breast cancer of cases and controls in the meta-analysis.

First author	Ethnicity	Case (genotype)			Control (genotype)				HWE
		A/A^a^	A/B	B/B	A/A	A/B	B/B	MAF	
ADPRT									
Yawei Zhang	Caucasian	1194	468	54	963	361	47	0.17	0.07
Xiangjun Zhai	Asian	100	153	49	197	331	111	0.43	0.16
Wen-hui Cao	Caucasian	65	17	1	72	28	0	0.14	0.10
Francine Durocher	Caucasian	40	13	1	53	19	1	0.14	0.62
Tasha R.Smith	Caucasian	236	71	7	272	114	11	0.17	0.81
Tasha R.Smith	African-American	46	6	0	69	3	0	0.02	0.85
APE1									
Yawei Zhang	Caucasian	404	752	373	327	590	290	0.48	0.45
Suleeporn Sangrajrang	Asian	250	208	49	194	176	55	0.34	0.13
Tasha R.Smith	Caucasian	103	140	76	104	209	92	0.49	0.50
Tasha R.Smith	African-American	23	22	8	30	33	12	0.38	0.56
K.Jelonek	Caucasian	16	50	25	90	223	99	0.49	0.09

a A represents the major allele, B represents the minor allele.

HWE: Hardy-Weinberg equilibrium; MAF: minor allele frequencies.

### Meta analysis

**Figure 2:**
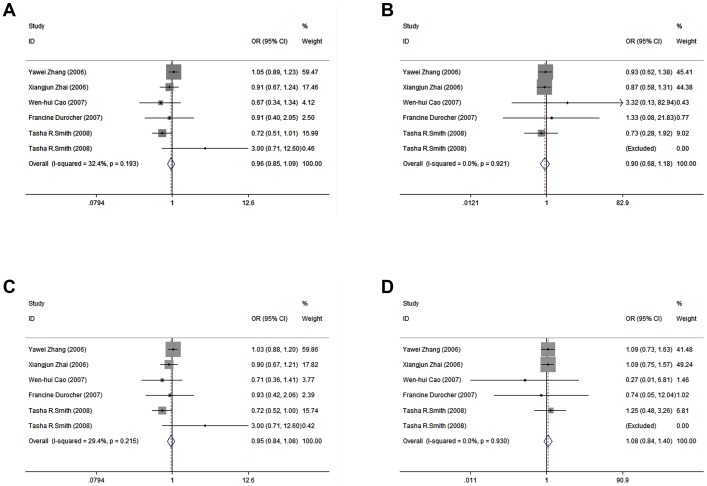
Forest plots for meta-analysis of the association between ADPRT Val762Ala polymorphism and breast cancer risk. A: Val/Ala vs. Val/Val; B: Ala/Ala vs. Val/Val; C: Dominant model; D: Recessive model.

As shown in Table 3, the results showed no significant association between ADPRT Val762Ala polymorphism and breast cancer risk (OR = 0.960, 95% CI = 0.845–1.090 for Val/Ala vs. Val/Val; OR = 0.897, 95% CI = 0.683–1.178 for Ala/Ala vs. Val/Val; OR = 0.953, 95% CI = 0.843–1.077 for dominant model; OR = 1.084, 95% CI = 0.838–1.403 for recessive model) (Figure 2). In the subgroup analysis by ethnicity or study design, the differences between the allele, homozygote, recessive, and dominant models were insignificant in the Caucasian women.

**Table 3:** Summary ORs and 95% CI of the association between polymorphisms in the two BER genes (ADPRT Val762Ala and APE1 Asp148Glu) and breast cancer risk.

Genetic model	No. of studies analyzed	Random-effects OR(95% CI)	Fixed-effects OR(95% CI)	Q	P for heterogeneity	I^2^
ADPRT						
Dominant model	6	0.916(0.762–1.102)	0.953(0.843–1.077)	7.08	0.215	29.4
Caucasian	4	0.891(0.710–1.118)	0.954(0.833–1.092)	4.48	0.214	33.0
Matched	4	0.929(0.735–1.175)	0.963(0.849–1.092)	6.34	0.096	52.7
Val/Ala vs. Val/Val	6	0.917(0.752–1.118)	0.960(0.845–1.090)	7.40	0.193	32.4
Caucasian	4	0.882(0.687–1.132)	0.959(0.833–1.103)	4.86	0.182	38.3
Matched	4	0.939(0.736–1.198)	0.974(0.854–1.110)	6.31	0.097	52.5
Ala/Ala vs. Val/Val	5	0.896(0.682–1.177)	0.897(0.683–1.178)	0.92	0.921	0.0
Caucasian	4	0.917(0.637–1.319)	0.919(0.640–1.321)	0.89	0.828	0.0
Matched	3	0.884(0.671–1.164)	0.883(0.671–1.163)	0.20	0.903	0.0
Recessive model	5	1.086(0.839–1.406)	1.084(0.838–1.403)	0.86	0.930	0.0
Caucasian	4	1.087(0.757–1.561)	1.083(0.755–1.553)	0.86	0.834	0.0
Matched	3	1.100(0.848–1.426)	1.100(0.848–1.427)	0.07	0.964	0.0
APE1						
Dominant model	5	0.928(0.788–1.093)	0.946(0.835–1.072)	5.38	0.250	25.7
Caucasian	3	0.955(0.717–1.271)	0.978(0.845–1.131)	4.67	0.097	57.2
Matched	4	0.946(0.751–1.192)	0.973(0.844–1.122)	4.77	0.190	37.0
Asp/Glu vs. Asp/Asp	5	0.928(0.779–1.106)	0.947(0.829–1.082)	5.42	0.247	26.2
Caucasian	3	0.928(0.670–1.285)	0.961(0.823–1.122)	5.29	0.071	62.2
Matched	4	0.922(0.707–1.201)	0.957(0.822–1.114)	5.35	0.148	43.9
Glu/Glu vs. Asp/Asp	5	0.944(0.779–1.144)	0.958(0.813–1.129)	4.55	0.337	12.1
Caucasian	3	1.018(0.849–1.221)	1.019(0.850–1.221)	1.86	0.395	0.0
Matched	4	1.013(0.848–1.212)	1.014(0.848–1.212)	1.94	0.585	0.0
Recessive model	5	1.004(0.873–1.154)	1.004(0.873–1.155)	3.15	0.533	0.0
Caucasian	3	0.959(0.825–1.115)	0.959(0.825–1.115)	0.35	0.839	0.0
Matched	4	0.962(0.829–1.116)	0.962(0.829–1.116)	0.40	0.940	0.0

**Figure 3:**
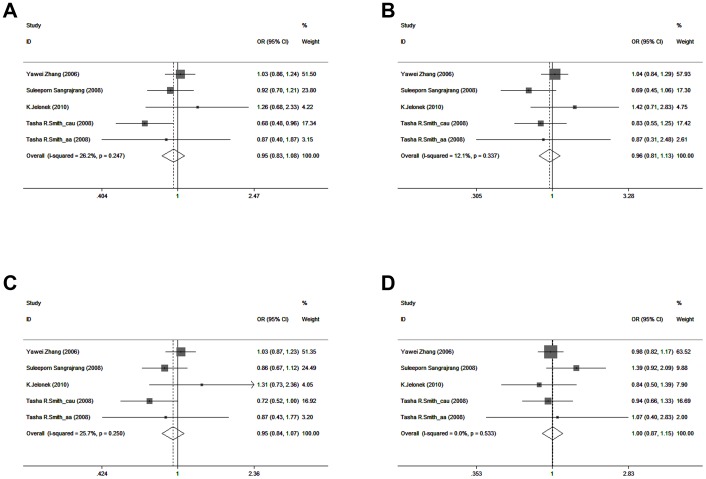
Forest plots for meta-analysis of the association between APE1 Asp148Glu polymorphism and breast cancer risk. A: Asp/Glu vs. Asp/Asp; B: Glu/Glu vs. Asp/Asp; C: Dominant model; D: Recessive model.

The associations between APE1 Asp148Glu and breast cancer risk are also shown in Table 3. The results indicated no relationship of APE1 Asp148Glu polymorphism with breast cancer risk (OR = 0.947, 95% CI = 0.829–1.082 for Asp/Glu vs. Asp/Asp; OR = 0.958, 95% CI = 0.813–1.129 for Glu/Glu vs. Asp/Asp; OR = 0.946, 95% CI = 0.835–1.072 for dominant model; OR = 1.004, 95% CI = 0.873–1.155 for recessive model) (Figure 3). In the Caucasians or the matched studies, no associations were found between the allele, homozygote, recessive, and dominant models.

### Sensitivity analysis

Sensitivity analysis was carried out by deleting any single study each time. The pooled ORs were not significantly altered (data not shown), indicating that the results were robust.

### Publication bias

**Figure 4:**
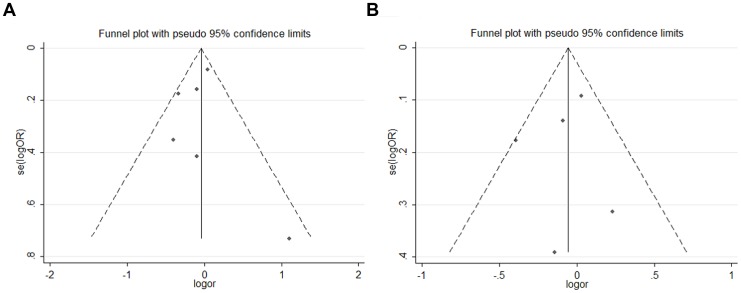
Funnel plot of publication bias for ADPRT Val762Ala and APE1 Asp148Glu polymorphism with breast cancer risk. Note: Funnel plot with pseudo 95% confidence limits was used. A: Funnel plot of publication bias for ADPRT (Val/Ala vs. Val/Val); B: Funnel plot of publication bias for APE1 (Asp/Glu vs. Asp/Asp).

Funnel plots and Egger's test were performed to assess potential publication bias of the literatures. The shape of the funnel plots showed that the dots nearly symmetrically distributed, predominantly within pseudo 95% confidence limits (Figure 4) and Egger's test suggested that no publication bias was detected in any comparison model (P>0.1).

## Discussion

An increasing number of articles on genetic association studies, genome-wide association studies (GWASs), and relate meta-analyses have been published to clarify the association between gene polymorphisms and breast cancer [22]. To the best of our knowledge, this is the first meta-analysis carried out to access the role of ADPRT Val762Ala and APE1 Asp148Glu polymorphisms in breast cancer. The findings suggested that the two BER pathway genes polymorphisms were not significantly associated with breast cancer risk. In subgroup analysis, no significant association was observed in sub-populations.

ADPRT plays an important part in DNA repair and cellular stress response. Its role in single-strand breaks through the BER pathway has been studied [22]. ADPRT Val762Ala substitution located within the COOH-terminal catalytic domain [23]. The functional relevance of this variant remains inconsistent. Several case-control studies showed significant associations between Val762Ala polymorphism and prostate and lung cancer risk [24], [25]. In contrast, others have reported that Val762Ala polymorphism was associated with reduced risk of non-Hodgkin lymphoma and squamous cell carcinoma [26], [27]. In this meta-analysis, we involved a total of 2,521 cases and 2,652 controls, no significant effects were observed between the allele, homozygote, recessive, and dominant models. The discrepancies between published studies might be due to different disease mechanism and/or carcinogen exposure in different populations, and study sample size. It is possible that ADPRT variant genotypes may be tissue-specific. Some studies have shown high or low ADPRT expression levels in different tumor tissues [28], [29], indicating ADPRT may play different roles in different types of tumors,

The APE1 Asp148Glu is the most extensively studied polymorphism in APE1. A study showed that APE1 Asp148Glu had no impact on endonuclease and DNA binding activities [30]. However, others have reported that the Glu allele was significantly associated with prolonged cell cycle delay in G2 phase and decreased DNA repair capacity after irradiation [31], [32]. Our results found no relationship between APE1 Asp148Glu polymorphism and breast cancer risk. In the subgroup analysis, significant risks were also not found among Caucasians and individually matched studies. One factor that would contribute to the discrepancy between different studies is that this polymorphism might play a different role in different cancer sites. A recent meta-analysis found a significantly increased risk of lung cancer among smokers in APE1 Glu allele carriers suggesting that there could be an interaction between cigarette smoking and APE1 Glu allele [33]. Another previous meta-analysis showed that the Glu allele may be a risk factor for colorectal cancer but not for other cancers, but the results should be explained with caution with limited sample size (3 studies for colorectal cancer) [34].

Some limitations might be included in the meta-analysis. First, although we collected all the eligible studies, the sample size of the included studies was not large enough, which could decrease the statistical power to better evaluate the association between the two gene polymorphisms and breast cancer susceptibility. Second, the overall outcomes were based on unadjusted estimates, while a more precise evaluation should be adjusted by other co-variants including age, body mass index, menopausal status, ethnicity, smoking status, alcohol consumption, and environment factors if individual data were available. Third, most of the included studies had conducted on Caucasians, and a few on Asians and Africans. Thus, more samples should be collected from Asians and Africans. Fourth, the genotyping method and the select criteria of controls in the studies were different. Finally, case-control studies with small sample size (<100 cases or 100 controls) might be reporting inflated ORs.

In conclusion, this meta-analysis suggests that ADPRT Val762Ala and APE1 Asp148Glu polymorphisms may not contribute to breast cancer risk. Large-sample studies of different ethnic groups with carefully matched cases and controls are needed to clarify the role of the two gene polymorphisms in the BER pathway and breast cancer susceptibility in the future.

## Supporting Information

Checklist S1 (DOC)
